# Dawn of a New Era for Membrane Protein Design

**DOI:** 10.34133/2022/9791435

**Published:** 2022-04-15

**Authors:** Shahin Sowlati-Hashjin, Aanshi Gandhi, Michael Garton

**Affiliations:** Institute of Biomedical Engineering, University of Toronto, 164 College Street, Toronto, ON, Canada, M5S 3E2

## Abstract

A major advancement has recently occurred in the ability to predict protein secondary structure from sequence using artificial neural networks. This new accessibility to high-quality predicted structures provides a big opportunity for the protein design community. It is particularly welcome for membrane protein design, where the scarcity of solved structures has been a major limitation of the field for decades. Here, we review the work done to date on the membrane protein design and set out established and emerging tools that can be used to most effectively exploit this new access to structures.

## 1. Introduction

Approximately one-third of all human proteins are located in membranes. However, due to the difficulty in solving membrane protein structures, they have received relatively little attention by the protein design community (see Table 1), as typical protein design pipelines require high-quality structures as a starting point. Of an estimated 6,700 membrane proteins in the human proteome [1], fewer than 200 structures have been solved [2]. This low representation is partly a result of low expression levels in native tissues [3]. For traditional protein structural determination methods such as NMR or diffraction-quality crystals, large and isolated amounts are needed. Proteins can be expressed using bacterial hosts such as E. coli; however, they require sophisticated systems that can selectively deliver membrane proteins to the correct cell membrane [4]. Additionally, due to the hydrophobic nature and lack of flexibility of membrane proteins, isolation methods with detergents generally result in aggregation as a result of misfolding or lower stability, decreasing the quality of solved proteins [3].

**Table 1:** Main functional categories of membrane proteins and their computational design examples.

Functional group	Type	Computational design examples
Receptor	GPCR	Allosteric signal transductions ([24]) Receptor-effector switches for rewiring signaling pathways [25] Computational design of thermostabilizing point mutations [26]
	Tyrosine kinase	tyrosine and serine kinase-driven protein switches [27] Design of a self-assembling symmetrical *β*-propeller protein [28]
	Cytokine receptors	Potent and selective mimics of interleukin-2 and interleukin-15 [29]
	Serine kinase	Tyrosine and serine kinase-driven protein switches [27]

Carrier/transporter	Channel	Transmembrane channel ([30]) Multipass transmembrane proteins ([31]) Nanopore for single-molecule detection [32] Channelrhodopsins [33]
	Carrier/transporter	Multicentered pathway for transmembrane electron transfer [34]

Enzyme		P450 2C3d engineered as soluble dimer [35]

Adhesion		N-cadherin [36]

A paucity of structures meant that a disproportionate amount of work over the last decade in membrane protein design has been performed using *de novo* approaches. *De novo* proteins are traditionally designed using two distinct methods—minimal and rational. A minimal design uses simple polar and hydrophobic rules to drive the design, while rational design uses sequence-structure relationships with computational tools to generate new backbone structures as templates. This is followed by fitting of sequences that are predicted to stabilize them. A *de novo* design of membrane proteins has been recently—and excellently—reviewed elsewhere ([5]) and will therefore only be lightly referenced in this review.

In 2021, however, there was a seismic shift in the protein structure field, precipitated by dramatic advances in machine learning-driven structure prediction. alphafold2 [6], developed by Google’s DeepMind, used an ingenious deep learning model to provide protein designers with reliable structure predictions for all proteins in the human proteome. In doing so, they imbued the protein design community with unprecedented potential for redesigning membrane proteins and thus designing human cells with new functions. Cell-based therapies are widely predicted to be the next major health care revolution [7], and this step in our ability to design human membrane proteins could contribute significantly to their development. Here, we set out the current state of development in using nature-derived membrane protein scaffolds for design and review established and emerging tools for capitalizing on the sudden wealth of high-quality predicted structures. Enhancing our ability to rationally design new membrane proteins is likely to have considerable impact in many areas of synthetic biology—particularly in sensing and engineering novel multicellular behavior.

### 1.1. Current Landscape of Natural Scaffold Membrane Protein Design

Early designs of membrane proteins can be traced back to the late 80s and were mainly based on the sequence and structure of known proteins. Based on the hydrophobic packing of amino acids facing the nonpolar acyl chains of lipids in the membrane, polar residues were employed to build the interior part of the channel [8]. This hydrophobic-hydrophilic alternating pattern (H2N-(Leu-Ser-Ser-Leu-Leu-Ser-Leu)3-CONH2) was employed to form ion-conducting channels. It was also shown that replacing a serine with leucine (H2N-(Leu-Ser-Leu-Leu-Leu-Ser-Leu)3-CONH2) would result in proton-selective channels. In another study, it was shown that the overall orientation of the protein in the membrane can be controlled by the positioning of lysine residues [9]. More sophisticated, rational structure-based design of membrane spanning proteins has proven highly challenging and is far less developed than the application of these techniques to water-soluble proteins. This is due to the lack of solved structures. For example, fewer than 400 beta-barrel membrane proteins (bBMPs) have been deposited in the protein databank and only ~60 of these are nonhomologous [9]. This provides extremely limited coverage of the estimated 70,000 bBMPs in nature [10]. Membrane proteins are typically classified by either their structure or function. Each structure type presents different challenges to protein designers. We will first examine the challenges posed by different structure types and then review the major functional types and how these functions have been modified.

### 1.2. Structural Classification

Membrane protein transmembrane structures are broadly divided into two categories: beta-barrel and alpha-helical. Alpha helix structures are the major class found in both prokaryotic and eukaryotic membranes. They include voltage-gated ion channels, G protein-coupled receptors (GPCR), and ATP-binding cassette (ABC) transporters to name a few. Helix-based transmembrane structures can be further subdivided into helical bundles and helical barrels. Bundles are relatively well characterized, while helical barrels have received much less attention [11]. The design of transmembrane helix bundles has received significant attention, from the identification of a motifs that direct helix-helix assembly and packing [12], to ligand specificity and intracellular signaling engineering of GPCRs [13, 14]. Interactions between transmembrane helices and their surrounding polar groups result in more stable helices in comparison to soluble proteins. Taking advantage of this property, a membrane hemoprotein, ME1, was redesigned with a glycophorin A to create a heme binding site [15]. Helix association propensity of amino acids in membrane proteins can be rank ordered (Gly > Ala > Val > Ile)—opposite of the trend observed for dimerization in water-soluble structures [16]. Stability and orientation of the transmembrane domain is thus mainly determined by noncovalent interactions, namely, van der Waals and electrostatic (including hydrogen bonds).

However, despite a long history, the design remains challenging. Even coiled coils, which have well-characterized packing rules and have been amenable to design in their water-soluble form for a couple of decades [17], are still largely refractory to the transmembrane design. A redesign of alpha-helical barrels using natural scaffolds is similarly underdeveloped. Examples of design include Cytolysin A—engineered to create a discrete lipid bilayer pore that can translocate DNA [18]. The design of active channels using short synthetic peptides is being energetically developed by the nanopore design community [19].

Membrane proteins are predominantly alpha-helical but transmembrane domains composed of beta-strands are also an important group. These are more commonly seen in bacteria, mitochondria, and chloroplast [20]. They commonly comprise a water-filled pore or solid core with polar side chains instead of a hydrophobic core. A potential term, EZ*β*, was determined for depth-related hydrophobicity to help redesign such proteins as they have very different structural features to other membrane proteins [20]. Most transmembrane beta-strand sequences alternate between polar and hydrophobic residues to create one hydrophobic face for the lipid membrane environment when folded. By adopting a beta-barrel structure, such that the hydrophilic face creates a polar interior channel, allowing transport of hydrophilic substrates. Transmembrane beta-barrels (TMB) range from 8 strands to 24 strands [21] and can function as both monomers and oligomers. The TMB design has the potential for many applications due to the strong substrate specificity and multiple control points. The design work on TMBs underwent a major recent development in the form of de novo design of experimentally validated transmembrane barrels that do not require assembly machinery or chaperones ([22]). However, the study was limited to 8-strand barrels. A design using a natural structural scaffold has been used to tune oligomerization states by introducing lipid facing strand mutations at protein–protein interaction sites [23].

A design using natural scaffolds has been greatly constrained by the lack of solved structures that represent a tiny fraction of known structures in nature. High-quality structure prediction now potentially allows access to the majority of known transmembrane structures. Using a combination of established and emerging computational methods, it should be possible to investigate the mechanics of these structures at the atomic level and gain a much broader understanding of the features that drive function. This knowledge, coupled with high-throughput screening, removes the major constraint to a transmembrane protein design.

### 1.3. Functional Classification

**Figure 1:**
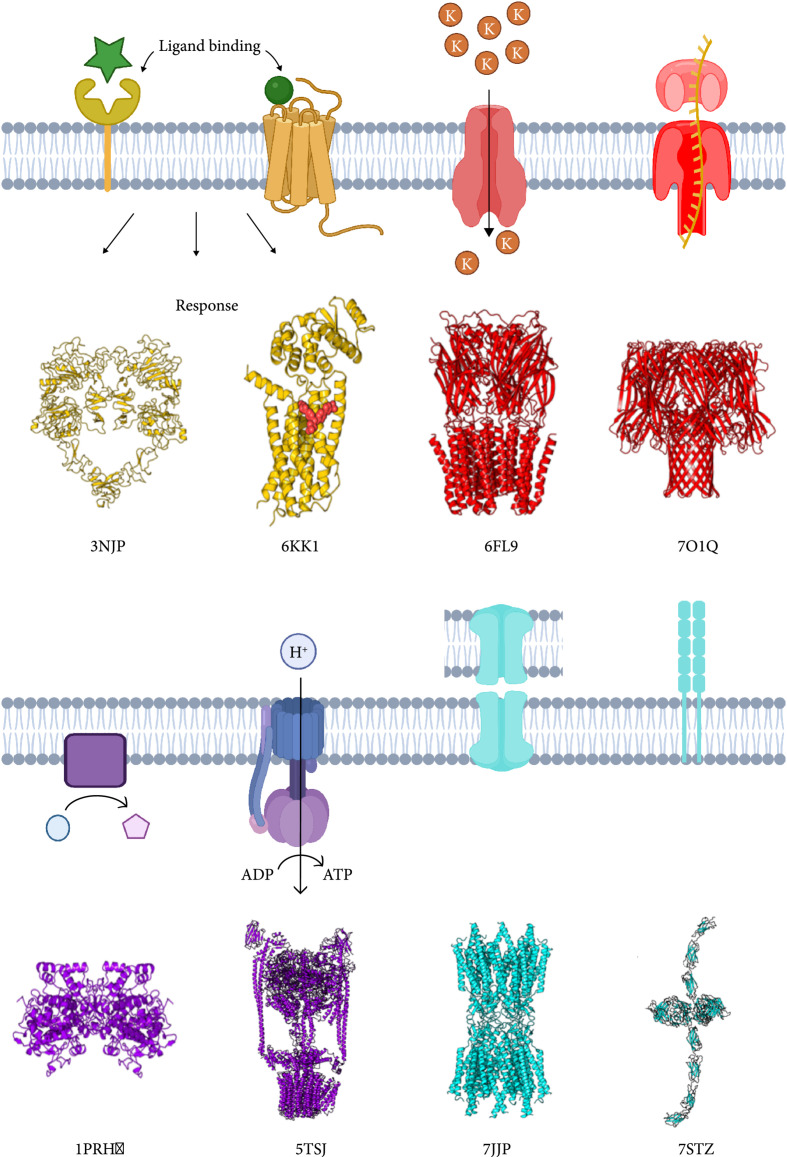
Schematic representation of membrane protein types and their structural examples. Receptor: tyrosine receptor (3NJP) ([37]) and GPCR (6KK1) ([38]). Transporter: potassium ion channel (6FL9) ([39]) and nanopore-hemolysin (7O1Q) [40]. Enzyme: prostaglandin (1PRH) ([41]) and ATP synthase (5TSJ) [42]. Adhesion: gap junction (7JJP) [43], E-cadherin (7STZ) [130]. Figures were made using Protein Imager [44].

Membrane proteins can be grouped into four major categories in the context of function and potential design. These categories are (1) receptors, (2) transporters, (3) enzymes, and (4) proteins involved in adhesion (see Figure 1).

#### 1.3.1. Receptor Design

Receptors have received the most attention in the context of design. G protein-coupled receptors (GPCRs) are the most important membrane receptors in eukaryotes, comprising approximately 35% of all drug targets [45]. They are activated via a diverse array of extracellular molecules that give rise to conformational change of the receptor and release of intracellular signaling molecules. These conformational changes occur in a variety of time scales. For example, retinal isomerization in rhodopsin takes place in a fs-ns time span, while GPCR-G complex dissociation requires seconds to minutes to complete [46]. Thus, a successful design of membrane receptors depends on a thorough understanding of the receptor mechanism of action and the associated structural flexibility, rearrangements, and the time scale necessary for such changes. The role of conformational changes and structural dynamics in function of GPCRs and Type-I transmembrane receptors has been comprehensively discussed in the literature ([47, 48]). There have been many successful attempts to coopt GPCRS as part of synthetic signaling systems. Designer receptors exclusively activated by designer drugs (DREADDs) are the most widely used [14]. Early designs were developed using simple site-directed mutagenesis to modify receptor affinity and selectivity. Later approaches employed directed evolution [49], and structure-guided rational design was also used to make more radical changes to GPCR activation mechanisms [50]. These design methods involve changes at the individual amino acid level—mirroring evolution by natural selection. These methods are distinct from traditional protein engineering approaches that typically fuse unrelated domains to produce chimeric proteins with new functions. Examples of receptors engineered using the fusion approach include chimeric antigen receptors (CARs) [51], synthetic notch receptors (synNotch) [52], modular extracellular sensor architecture (MESA) [53], and transcriptional activation following a resting translocation (TANGO) receptors [54]. CAR and MESA receptors have also been refined and optimized using structural modeling and computational docking [55, 56]. However, due to the lack of solved structures with similar sequences, the development of more variants has been limited.

#### 1.3.2. Transporter Design

Transporters are involved in the movement of a wide variety of molecules across membranes. These include ions, small molecules, and macromolecules such as proteins and nucleic acids. Transport is driven by facilitated diffusion or active transport. From a design and engineering perspective, ion channels have received by far the most attention. This has been largely motivated by a desire to better understand how they function, but also to probe cell biology and physiology [57]. Several studies have adapted ion channels for use as biosensors. For example, green fluorescent protein (GFP) was inserted/fused with an intracellular loop of a voltage gated sodium channel to produce fluorescent signal in response to membrane potential changes [58]. These biosensors are called sodium channel protein-based activity reporting constructs (SPARCs). A huge range of light-activated and ligand-activated ion channels/pumps have also been developed and deployed across a broad range of applications, most notably precipitating seminal advances in neuroscience [59]. In the past few years, there have been promising advances in sensing and sequencing using engineered protein nanopores. Specifically, nanopores have been designed to transport ions across the membrane ([30]) sequence homonucleotides with high fidelity (Van [60]), to read single proteins at single-amino acid resolution [61], and proteasome-nanopores to process single proteins [62].

#### 1.3.3. Membrane Enzyme Design

Membrane enzymes catalyze important chemical processes such as methylation, drug metabolism, ATP synthesis, and hydrolysis. Understanding how enzymes catalyze reactions in membranes is still extremely limited, due in large part to the lack of structures. A systematic search of the literature yields little to no examples of the membrane enzyme design, beyond deletion of the P450 enzyme membrane anchor domain to produce a soluble/secreted version of the enzyme [35].

Challenges with designing membrane proteins are even more profound in the case of membrane enzymes, considering the competition between cavities for enzymatic activity (active site) and tight packing for thermodynamic stability of the protein [63] as creating a cavity could destabilize the enzyme due to the loss of packing. Thus, engineering membrane enzymes require fine-tuning the function and stability of the cavity. In a recent experimental and computational study, a cavity-filling mutation approach was employed to elucidate conformational changes regulating the function of GlpG, where cavities were shown to be stabilized by interactions with membrane lipids and hence balance between stability and function [63]. This strategy could thus be used to engineer membrane enzymes.

#### 1.3.4. Adhesion Protein Design

Cell adhesion molecules are primarily involved in mediating the interaction between a cell and its neighbors or the extracellular matrix. These proteins are crucial for maintaining tissue structure and function. An early design of adhesion proteins involved rational design to enhance affinity between integrin lymphocyte function-associated antigen-1 (LFA-1) and its ligand: intercellular adhesion molecule (ICAM-1) [64]. More recently, cadherin variants have been designed using computational modeling and validated experimentally as having affinity increased or decreased by up to 2 orders of magnitude [36]. Adhesion proteins often have large extracellular or cytoplasmic domains that can be purified independently of the transmembrane domain and their structures readily solved. Design involving these regions has therefore historically been much more accessible.

## 2. Structure-Guided Transmembrane Protein Design Tools

A number of useful tools are available to broaden and enrich our understanding of transmembrane protein mechanics from structural investigation. These will be critical for processing the vast number of new protein structures predicted by alpha-fold. Previous examples of redesigning membrane proteins with computational approaches have shown the potential for tuning interactions within membrane proteins and introducing new functionalities with established protocols [46]. In this section, recent advances in computational structural analysis and design with respect to membrane proteins are reviewed and emerging techniques that could be coopted are discussed.

### 2.1. Membrane Protein Molecular Dynamics Simulations

Molecular dynamics (MD) simulations of membrane proteins are generally computationally more resource intensive due to the requirement for phospholipid molecules (lipid bilayer membrane). Until very recently, multimicrosecond MD studies on such systems were only feasible using a coarse-grained approach. Currently, microsecond-long MD simulations on large protein-membrane systems (10^5^-10^6^ atoms) are achievable in the span of weeks. In this section, previous MD studies on the structure and function of select membrane proteins are reviewed.

### 2.2. MD Simulation Setup for Membrane Protein Systems

Modeling of membrane proteins requires protein embedding in a lipid bilayer. The VMD package [65] can be used to set up such systems for the NAMD simulation package [66]; nevertheless, its database for phospholipids is limited to only POPC and POPE. GROMACS [67] is also capable of building a membrane-protein system for MD simulations and requires force field parameters to be added for any given lipid molecule. However, CHARMM-GUI ([68]) is probably the most versatile, user friendly, and automated tool for MD simulation input preparation, not only for membrane and membrane protein systems but also for soluble proteins, polymers, nanodiscs, nanomaterials, micelles, monolayers, etc. To our knowledge, CHARMM-GUI provides the most comprehensive set of parameters for lipids required for modeling a variety of membranes and generates inputs for most of the widely used MD packages such as CHARMM, Amber, Gromacs, NAMD, and Desmond, using CHARMM, AMBER, and OPLS force fields. It also provides options for modification of N- and C-termini, protonation states of ionizable amino acids, set up disulfide bridges, and mutations during the early steps of the system setup. One particular challenge in the preparation of membrane protein systems is the orientations of proteins in the membrane. To address this issue, the OPM database [69] has been developed. This database includes more than 1,200 membrane proteins representing about 3,800 Protein Data Bank entries. Considering the hydrophobic and electrostatic interactions of the proteins with both water (polar) and lipid (nonpolar) environments, the PPM 2.0 and 3.0 (positioning of proteins in membranes) [70] optimize the positions of protein within the lipid bilayer. CHARMM-GUI uses this database and PPM server and automatically sets up the protein in the lipid bilayer of interest.

### 2.3. MD Studies of Membrane Proteins

MD simulations on membrane proteins have been employed to study a wide variety of systems and properties such as structural analysis of the protein in lipid environment [71], linking structure to function [72], oligomeric state of membrane proteins [73], role of specific amino acids in the function [74], transport function through change of state [75], permeation [76], and selectivity mechanisms [77], to name a few. For a detailed evaluation of simulation protocols for membrane proteins, see reference [78]. In this section, we highlight a few notable membrane protein MD studies.

#### 2.3.1. Case Study #1: Conformational Changes in ABC Transporter

ABC transporter facilitates import-export of ligands across the membrane using adenosine triphosphate (ATP). The translocation of a ligand mediated by ABC requires a large-scale conformational change in the transmembrane domains (TMDs), converting the cytoplasm open-state to the periplasm open-state and vice-versa. The free energy required for such a process is provided by the ATP binding, hydrolysis, and dissociation of the product at the nucleotide-binding domains (NBDs).

**Figure 2:**
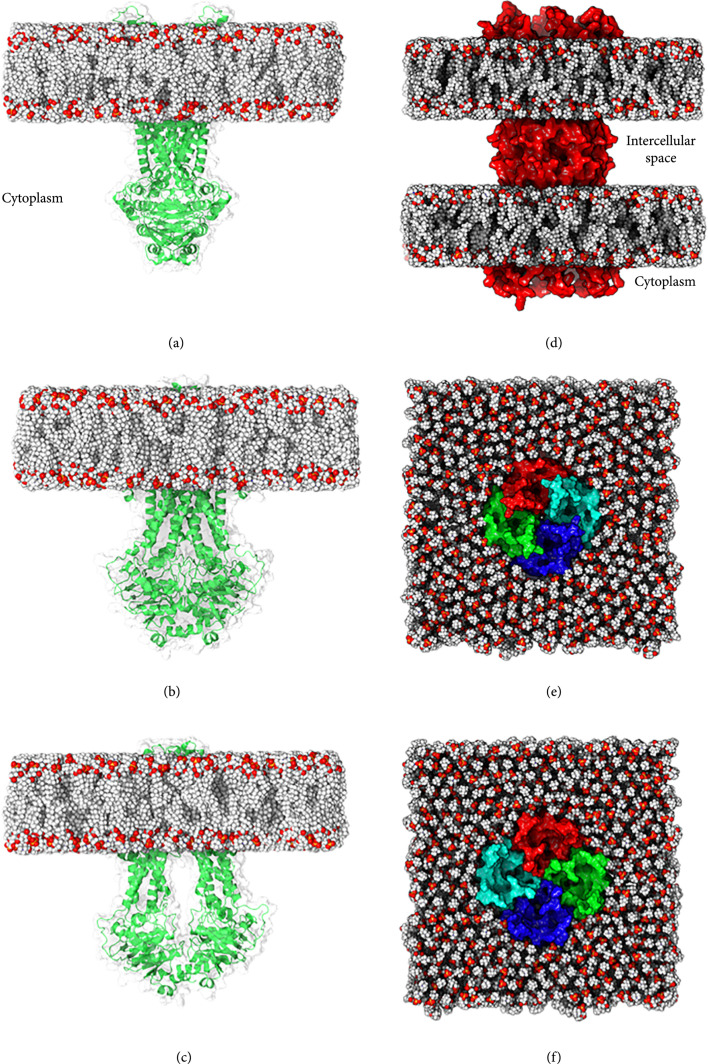
Examples of membrane protein structures in lipid membranes. (a–c) Three states of ABC transporter (a) outward-facing, (b) occluded, and (c) inward-facing structures. (d) Structure of a gap junction (Cx50, PDB ID: 7JJP) embedded in two lipid bilayers. (e) Extracellular and (f) intracellular views of aquaporin-1 (AQP1) [81].

ABC transporters have been the subject of several computational and computational-experimental studies ([75, 79, 80]). A comprehensive multimicrosecond-long MD (100 simulations of at least 500 ns length) study combined with electron paramagnetic resonance (EPR) were employed to elucidate the conformational transitions of the heterodimeric ABC exporter (TM287/TM288) [79]. It was shown that the protein’s state changes from the cytoplasm open-state to the periplasm open-state through an occluded intermediate. The occluded and periplasm open-state structures obtained with MD simulations were in excellent agreement with the corresponding crystal structures. The transport mechanism elucidated by the MD simulations involves cooperative motions of both transmembrane and nucleotide-binding domains. The occlusion state ensures that the cytoplasm and periplasm sides are not simultaneously open (Figures 2(a)–2(c)). This study reveals that while vital for the function, conformational changes required for switching between the states involves a considerable energy barrier to overcome.

#### 2.3.2. Case Study #2: Selectivity Mechanism in Aquaporins

Aquaporins and aquaglyceroporins are a family of integral membrane proteins that function in a tetrameric form and are responsible for the selective translocation of solutes across the cell membranes. In a comprehensive MD study, the selectivity and permeability of human AQP1 (hAQP1) (Figures 2(e) and 2(f)) and GlpF as members of the two aquaporin subfamilies for some small molecules including O_2_, CO_2_, NH_3_, glycerol, urea, and water were examined [81]. In order to obtain the permeation barrier across the protein, the potential of mean force (PMF) was calculated using the umbrella sampling technique for all the solutes. For a detailed overview of the process, see references [82, 83]. From the MD results, a selectivity site for uncharged/nonpolar solutes in both aquaporin and aquaglyceroporin was identified (called ar/R). This region is narrow and hydrophilic in hAQP1. The interaction between water molecules and polar amino acids in this region can be replaced by more favorable interactions between the amino acids and a polar solute. However, larger molecules such as urea and glycerol are also filtered through steric clashes they cause with this narrow passage. The ar/R site is wider in GlpF and also includes more nonpolar amino acids, which makes GlpF permeable to a broader range of molecules such as polyols. It was suggested that the permeability of AQP1 is controlled by a rather simple filter mechanism, where large molecules are excluded through steric occlusion and the ar/R region is selective for small and hydrophilic molecules. GlpF on the other hand is less selective and can effectively translocate all molecules considered in the set except for urea. It was concluded that water–pore (and not solute–pore) interactions combined with steric effects control the selectivity and the energy barriers in AQP1 and GlpF.

#### 2.3.3. Case Study #3: Oligomerization of NanC

It is estimated that ~25% of the cell membrane is occupied by membrane proteins [84]. Such a crowded environment promotes the oligomerization of membrane proteins; however, it has been shown that lateral interactions between the proteins are not solely driven by the lipid raft [85]. Oligomerization has been reported for many membrane proteins [86]. In fact, oligomerization motifs have been identified in several membrane proteins [87]. The lateral interactions and dimerization pattern of membrane proteins have been investigated for a bacterial outer membrane protein, NanC, responsible for transportation of sialic acid. With the aid of coarse-grained MD simulations and umbrella sampling technique, association energy landscape of two NanC monomers with four different orientational configurations were examined in a lipid bilayer [88]. The obtained energies range between -66 and -45 kJ/mol, depending on the shape complementarity of the proteins. It was also shown that the PMF shows nearly linear dependency to the buried surface area.

#### 2.3.4. Case Study #4: Redesigning FhuA Nanochannel

Redesigning and engineering of proteins to enhance and/or alter their function is of great interest. For example, separation of racemic mixtures into pure enantiomers has been shown to be carried out by reengineered proteins. Specifically, a redesigned beta-barrel membrane protein, FhuAF4, derived from FhuA, was shown to be able to resolve D- and L-arginine mixtures [89]. Steered MD simulations (SMD) were used to find the molecular basis of the enantiopreference of the FhuAF4 for L-arginine. It was shown that while D-arginine interacts with two residues (S134 and T146) in the selectivity filter region of the FhuA4, L-arginine freely passes through the channel and only forms hydrogen bonds with S134. Thus, the obtained preference of FhuA4 for L-arginine is a result of its ability to form more interactions with D-arginine.

#### 2.3.5. Case Study #5: Rational Design of Aerolysin Nanopores

In a recent study, a set of mutated aerolysin nanopores were rationally designed for detection of a range of biomolecules [90]. It was revealed that the selectivity and sensing function of the aerolysin is governed by electrostatic properties of the pore as well as the pore diameter. It was shown that the pore diameter could be adjusted (from 0.5 to 1.5 nm) by site-directed mutagenesis. This in turn, allows for detection of a broad range of molecules by aerolysin. To validate the models, MD simulations were carried out on the aerolysin pores, and the ion current across the pore was calculated. The electrostatic potential at 100 mV voltage was calculated and compared to those obtained experimentally. The measured pore current was shown to agree with MD prediction, hence proving the molecular explanations.

With the invention of alphafold and recent advances in both computer hardware and MD simulation algorithms, it is expected to witness a new wave of MD studies on the membrane proteins in the next few years.

## 3. Channel Characterization Tools

Channel proteins govern many physiological functions and allow for transportation of moieties necessary for cell survival. Depending on their function, the physical properties of membrane proteins vary and channels assume different states to control the transportation function. For example, permeation in ion channels is controlled via energy barrier regulation or gates along the pore. Of particular interest are the physicochemical properties of channels among the different families of membrane proteins. Some important properties include (but not limited to) channel width and length, hydrophobicity, hydropathy, polarity of lining amino acids, lipophilicity, solubility, and water number density. Traditionally, the channels were examined by size-based approaches, which provides useful information about the permeation pathway. However, gates that are not based on steric occlusion of the pathway cannot be identified by this method. In this section, some of the available and more widely used tools for the analysis of membrane protein properties, including channel properties, will be introduced.

### 3.1. HOLE

Developed based on the first pore analysis study ([91]), the HOLE program was designed to analyze and visualize the holes along the ion channels and was introduced in 1996 ([92]). HOLE descriptions can be found at http://www.holeprogram.org/, and the source code is freely available to download at https://github.com/osmart/hole2. The program was developed for single structure analysis and is not designed to process MD simulation trajectories, where sequential reads and processing is required. HOLE accepts PDB format and to initiate the analysis requires the user to specify an initial point along the channel as well as a vector that represents the direction of the channel. A HOLE run on a PDB structure provides pore radius of the protein, which can be illustrated in different ways, including a 2D plot of pore radius against distance along the channel and a 3D representation of the channel. HOLE is also capable of estimating the conductance of a channel based on the protein structure.

### 3.2. PoreWalker

**Figure 3:**
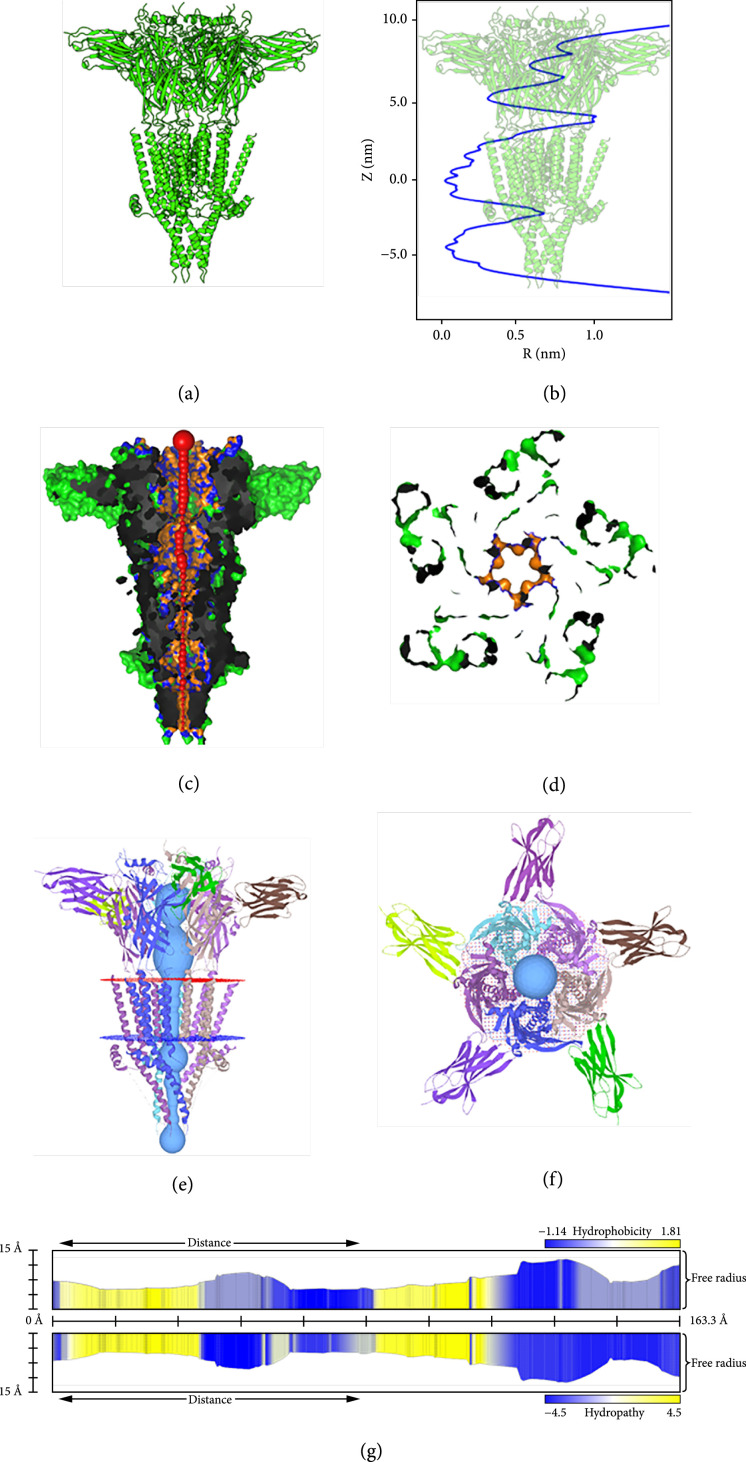
Output examples of membrane protein analysis tools. (a) Structure of 5-TH3 receptor (PDB ID: 4PIR) ([97]). (b) Radius profile produced by CHAP. (c) Pore represented by red spheres proportional to the diameter by PoreWalker. Blue and orange represent pore lining residues and atoms, respectively. (d) Cross-section of the protein (PoreWalker). (e, f) Side and top views of the channel. (g) Pore properties (free radius, hydrophobicity, and hydropathy calculated along the pore by MoleOnline). While visualization methods and outputs vary for these methods, they all demonstrate the nature of the channel and capture its important features. It is recommended that the user carefully reads the documentations of each program and decides which one may be most suitable for a particular system.

PoreWalker [93] is an automated tool that identifies and characterizes transmembrane protein channels. It only accepts a single structure in PDB format. The program takes the following four steps to analyze the pore properties: (i) determination of the main axis of the protein, (ii) optimization of the center of the pore, (iii) identification of the best cavity and pore axis optimization, and (iv) analysis of the channel properties including size, shape, and identification of pore lining amino acids. Once the analysis is completed, PoreWalker provides the outputs to download including channel shape and width and pore lining residues. The output files also include side view and top view cross-sections of the channel (Figures 3(c) and 3(d)).

### 3.3. MOLEonline

The updated version of MOLEonline [94] is a free web-based tool released in 2018 and is designed to analyze two modes: channels and transmembrane pores. It accepts both PDB and mmCIF formats and is capable of extracting the PDB structures automatically from the PDB database if provided with the PDB ID. The pore mode computation, which is more relevant to the subject of the current review, involves a membrane domain definition by the aid of the Orientations of Proteins in Membranes (OPM) database (http://opm.phar.umich.edu/) or the MEMEMBED [95] program calculations. The planes defining the top and bottom leaflet boundaries are used to define the initial and final points along the normal near the center of the protein. To characterize the pathway properties, default MOLE parameters can be used; however, the program allows the user to modify the cavity parameter values such as *probe radius* and *interior threshold*, which control the alpha-shape surfaces and beta-shape cavities of the protein, respectively. MOLEonline uses an interactive graphical interface and allows the user to select a property of interest to be illustrated along the pathway. These properties include charge, hydrophobicity, polarity, lipophilicity, solubility, and mutability. MOLEonline employs three different radius definitions to characterize the width of the pore, namely, radius, free radius, and BRadius. Radius of pore is defined as a sphere within the pore limited by the three closest atoms, while free radius calculation uses the three closest backbone atoms, which allows for sidechain flexibility. To include the local flexibility of the residues, BRadius calculates the RMSF of the amino acids from the B-factors included in the structures and adds it to the calculated radius (Figures 3(e)–3(g)).

### 3.4. CHAP (Channel Annotation Package)

Channel annotation package (CHAP) was introduced in 2019 and is freely available. CHAP is written in C++ language and unlike HOLE is capable of MD trajectory analysis. CHAP can be run on PDB structures directly and provides radius and hydrophobicity profiles. Additional information such as water number density and free energy profiles require performing MD simulations. The resulting MD trajectories are used by CHAP through a workflow to estimate and produce such profiles. These steps are comprehensively illustrated and discussed in the original paper [96]. In this section, only a brief and simplified overview is provided. After defining the physical dimensions of the pore, similar to HOLE, a probe-based method is used to obtain a sequence of discrete probe positions, which provides the associated pore radii (Figure 3(b)). This will be later converted to a continuous spatial curve using B-spline interpolation. Interpolating the radii along the obtained curve will then produce a continuous radius profile. In the next step, pore lining residues are used to determine the hydrophobicity profile of the channel. The discrete values obtained for each amino acid are converted to a continuous hydrophobicity profile. To estimate the water density along the channel, CHAP transforms the water molecule positions to channel coordinates, which is used to calculate a one-dimensional probability density. Then, the channel’s radius profile and water probability density are used to calculate the number density. Finally, the Boltzmann inversion is employed to estimate the solvent free energy profile along the channel. The whole process will be repeated for each individual structure if there is more than one frame (such as when an MD trajectory is used as the input file).

## 4. *In Silico* Protein Design Tools

Computational protein design tools typically involve backbone sampling, scoring functions, sequence optimization, and functional site design [98]. Naturally existing protein function is dependent on the stability of its backbone, which dictates the shape. Optimizing sequences of naturally existing proteins generally try to maintain backbone conformation and select compatible amino acid side chains. These methods, most notably Rosetta and FoldX, include scoring functions which rank designs based on sequence-structure combinations, energy terms, and statistical-based methods from known structures. Applying these techniques to membrane protein design, it has been shown that synthetic membrane proteins can be generated that fold and have good stability ([31, 98, 99]). They are able to accurately predict membrane compatible orientations of amphipathic aromatic residues with positively charged residues on the cytoplasmic side [98]. Scoring membrane proteins has been particularly challenging due to the lack of previously solved transmembrane proteins in the Protein Database Bank. The huge number of new predicted protein structures is now available may lead to significant improvements in accuracy.

### 4.1. Calculating the Free Energy of Binding

Free energy calculations can be used to accurately predict the contribution of individual amino acids to protein function and as such can be used to assist protein design. Thermodynamic integration (TI) and free energy perturbation (FEP) are currently among the most accurate computational techniques for free energy calculation. They have been consistently shown to match experimental binding free energy values with minimal error [100–102]. Drug–target, protein–protein [103], protein–DNA [104], and protein– peptide [105] interaction energies have all been accurately predicted using TI. Until recently, performing anything more than a very small number of TI calculations was prohibitively resource intensive. Usually, it was necessary to run very short simulations and severely limit the number of replicates. However, the recent arrival of GPU accelerated TI [106] has dramatically increased capabilities in this regard. It is now possible to test dozens of mutations over significant time scales and perform many replicates. Similarly, fast growth TI (FGTI) [107] has been shown to provide reliable results. Given these advances, it should now be possible to calculate protein–protein binding affinities between two membrane proteins, though this has yet to be demonstrated. Application of these highly accurate techniques to membrane proteins would allow high confidence characterization and design.

It is timely to emphasize that while binding affinities for protein–ligand and protein–protein systems can be experimentally determined for soluble proteins, in many cases, membrane protein systems do not possess soluble parts and lipid membranes are required for their stability and function and hence experimentally determining the binding affinity is not possible with current methods.

### 4.2. Design Using Artificial Neural Networks

Machine learning and particularly deep learning are leading to major breakthroughs across a broad range of sectors, particularly image classification and natural language processing. These successes have now been extended to solving major challenges in computational biology, alphafold being perhaps the most significant to date. Protein design using generative models such as generative adversarial networks (GANs) are still a very nascent field, with just a handful of experimental validated methodologies published [108–110]. It should be noted that none of these methodologies claim to generate new or improved proteins, but simply demonstrate that new polypeptide sequences can be generated that successfully fold and function on a par with their wild type counterparts. Generative models are typically trained on multiple sequence alignments of thousands of protein variants. Variants are typically evolutionarily related orthologs and/or from deep mutational scanning. The latter has the advantage that negative data can be used to train. It is certainly feasible that these techniques can be employed in the generation of new membrane proteins. Combining generative models with alphafold-derived structure prediction is likely to be especially powerful.

## 5. Integrating Computational Design with High-Throughput Functional Selection

### 5.1. Directed Evolution

**Figure 4:**
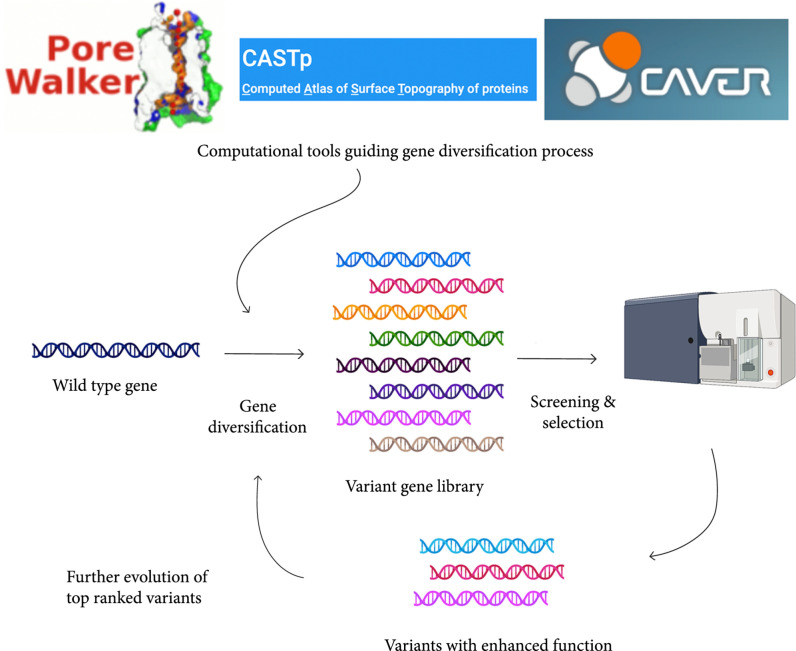
Schematic illustrating how directed evolution works to create variants that have desired functional changes. Wild-type gene undergoes gene diversification which can be facilitated with computational tools guiding the types of amino acids being mutated at each position. These variant libraries then are screened and selected for using high-throughput assays such as fluorescent-associated cell sorting (FACS). The variants showing the desired functional change then undergo evolution again until the designs are isolated as the most effective design

Directed evolution is done by diversifying the native protein sequence and filtering the designs with the most promising function ([111]). Diversification methods can occur through random mutation or by targeted approaches (see Figures 4). Instead of mutating the entire protein sequence which generates an unmanageable number of variants, focused mutagenesis can be used to limit sequence space to library sizes below commercially synthesizable limits [112]. Knowledge of the structure is invaluable for determining which residues to vary and may also allow informed restriction of residue type—both of which help to minimize sequence space. In addition to some of the characterization methods mentioned above (HOLE, PoreWalker, etc.), there are other computational methods such the computed atlas of surface topography of proteins (CASTp) that have been used to guide rational design. This software identifies pockets and voids in 3D protein structures for ligand binding, DNA interaction, and enzymatic activity (Yajie [111]). Another computational tool, CAVER 3.0 calculates and clusters tunnels and channel pathways to characterize individual pathways, their time evolutions, and gating mechanisms [113]. A more in depth review describing many other computational tools for structural features was recently published [114]. An example of computational design-driving protein design is the acetylcholine receptor alpha-1 subunit, a ligand-gated ion channel which was redesigned to be a water-soluble protein allowing structurally characterization with NMR [115].With the information obtained from these tools, protein design regions can be carefully selected according to morphological, geometrical, and chemical constraints. For instance, restricting design for only hydrophobic residues in membrane protein’s core would allow a manageable library size that is tailored for the specific application.

For membrane proteins specifically, directed evolution has been used to improve GPCR stability in detergents and increased expression levels—the two limitations affecting membrane protein studies [116]. A library of neurotensin receptors (NTR1) was created with error-prone PCR, induced within the inner membrane of *E. coli*, and introduced with fluorescent agonists to select variants with maintained binding affinities [116]. These were isolated with fluorescence-activated cell sorting (FACS) and were rediversified and screened for four rounds. Using computational tools to optimize sequence design of a poor resolution structure, statistical probabilities were calculated to limit residue mutations at each position [116]. This method involves ex mammalian systems where the proteins are purified and evolved *in vitro.* Since purification and production of membrane proteins remains a challenge, methods that can be done within mammalian systems offer a great advantage. Such an example is the viral evolution of genetically actuating sequence (VEGAS) system. This uses the RNA alphavirus Sindbis, to link viral vector replication with the diversification of the target protein under selective pressures [49]. Their proof of concepts showed the ability to evolve tetracycline transactivator (tTa) to avoid inhibition by doxycycline, GPCR to activate endogenous signaling pathways, and nanobodies to selectively activate GPCRs to activate serum response [49]. Other mammalian-based methods such as TRACE [24] and CRISPR-X [117] rely on cytidine deaminase which introduces point mutations by deaminating cytosine to uracil resulting in three different mutagenic processes, known as somatic hypermutation (SHM). CRISPR-X uses dead Cas9 (dCas9) and a single guide RNA (sgRNA) with hairpin-binding sites to recruit MS2 proteins fused with AID∆ to initiate hypermutation [117]. They were able to show this system evolved spectra-shifted variants of GFP and mutated PSMB5 against its inhibitor, bortezomib. However, this method is limited by where the sgRNA binds to; therefore, there are tight genomic windows where mutations can occur. TRACE was developed with this limitation in mind and inserts T7 promoter and uses a T7 RNAP linked with a cytidine deaminase to generate mutations 2 kb downstream of the promoter [118]. They showed this system to shift the fluorescent spectra of blue fluorescent protein to GFP and also evolve mitogen-activated protein kinase 1 (MAP2K1) to be resistant to pharmacological inhibitors [24]. Although these systems have not been used to evolve membrane proteins, we see no reason for future work not to be applied with these methods. Additional systems for directed evolution in mammalian cells that are not specific to membrane proteins are captured in this review [119].

**Figure 5:**
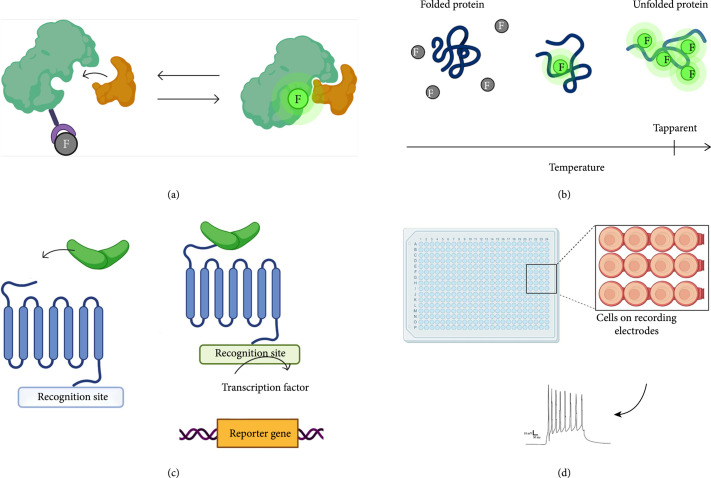
Graphical representation of the high-throughput methods. (a) Fluorescent-based assays that use a probe that fluoresces upon interaction with target protein of interest. (b) Thermal scanning assays use the natural denaturing of proteins to expose binding sites which fluorescent probes bind to. Using the change in fluorescence signal, the apparent temperature can be determined which provides insight on the stability of the designed protein. (c) Gene expression assays use a recognition site that is coupled with a transcription factor. Upon ligand binding, the recognition site triggers the transcription factor to activate downstream reporter genes. (d) Automated patch clamp methods allow for screening of 384 cells in parallel capturing individual electrical activity in a high-throughput manner

After successful diversification, libraries of up to a billion gene variants can be generated, and high-throughput screening strategies can be used to select for functional variants ([111]). Functional assays that can be performed as high-throughput screens allow for tractable evaluation of these vast libraries (see Figures 5). To achieve the most accurate results, membrane proteins should be tested in an environment closest to that of the final application. Common selection methods are discussed below and are grouped based on similar mechanisms, i.e., fluorescence based, thermal scanning, and electrophysiology assays.

Fluorescence-based assays work by measuring the emission of fluorophore upon interaction with the protein target of interest. This method of detecting protein–protein interactions and ligand binding has high sensitivity, is noninvasive to the sample, and has relatively fast response times [120]. Functions of transporters such as ion channels have also been characterized using an indirect measure of membrane potential or ion concentration changes based on fluorescence changes [121]. In recent years, the introduction of voltage-sensitive dyes and ion-specific fluorescent probes has increased sensitivity and ability to screen variants in a high-throughput manner. Voltage-sensitive dyes are enzyme substrates designed to fluoresce upon enzymatic cleavage in cells [122]. Fluorescent probes have been developed to be ion-specific in order to quantify the channel activity [121]. One specific example is the FluxOR assay which uses thallium-affinity probes that are activated as extracellular thallium enters the cell through potassium channels and the signal is interpreted on a plate reader [123]. This serves as an indirect measure of potassium channel activity and is able to highlight inhibitors of the potassium channel. Fluorescence-based assays can also be used to characterize enzymatic membrane protein functions such as the one developed in 2019 [124]. Using apolipoprotein n-acyltransferase transferred onto biotin and click chemistry to conjugate a fluorescent group to the fatty acid, they were able to show product formation using fluorescent signals with the presence of enzymes compared to without. Previously, fluorescence polarization was used to study interactions between proteins and molecules. This is a binding assay which uses the change in emission light when a fluorescent molecule is unbound and bound to a larger molecule [125]. This has been used in the past to discover inhibitors and activators for GPCRs [126].

In addition to functional changes, designed protein stability is also important to evaluate. One method to measure stability is thermal scanning which measures protein unfolding with a fluorescent marker. To integrate this approach as a high-throughput screen, GFP-thermal shift assays were used to quantify ligand binding to solute carrier (SLC) transporters [127]. A method in 2009 used thermal scanning with lipophilic dyes to bind to the protein in the unfolded state and thus fluoresce at room temperatures [128]. This method can be used to indirectly measure the strength of protein–ligand binding based on which temperature the fluorescence signal peaks—indicating when the protein has unfolded. MaMTH luciferase interaction assay is a split-ubiquitin assay that uses a transcription factor release to drive expression of luciferase or GFP [129]. Another example of this is the TANGO assay which links GPCR activation to TEV protease cleavage of its cognate site and consequent release of a transcription factor to the nucleus to activate reporter gene expression [13]. To use either assay, the C terminus of the target protein gets fused with the recognition site (ubiquitin or the TEV cleavage site) and its corresponding transcription factor.

Electrophysiology methods allow for the tracking of ions through voltage-sensitive channels, and the historical gold standard is the patch-clamp method. More recently, automated electrophysiology assays have been developed that offer a promising alternative. IonWorks and its derivatives have shown success in single-cell recording along with population patches. The SyncroPatch 384PE is another device that can clamp 384 cells in parallel to record around 20,000 data points a day [103] Taken altogether, these high-throughput methodologies offer the membrane protein designer an array of effective tools to screen variant libraries generated using structure-guided computational design.

## 6. Conclusions and Outlook

Transmembrane protein design holds much potential for a broad range of advancements in synthetic biology and biotechnology. Despite decades of development however, the field is still woefully underexplored. This has almost entirely been due to the paucity of solved structures, meaning that rules of assembly and packing have been difficult to elucidate. An absence of natural scaffolds and design rules meant that large swathes of membrane protein types have received little to no attention by protein designers. With the recent advent of high-quality structure prediction in the form of alphafold, this barrier has been very significantly lowered. The field is now poised to systematically investigate and elucidate structure and function at the atomistic level. To do this, a range of established and emerging tools are at the disposal of investigators in the field. Deploying these tools to dynamically model and characterize predicted structures will yield an unprecedented number of natural scaffolds for design. It is likely that the structural coverage engendered by systematic investigation of predicted structures will provide membrane protein assembly insights that will further catalyze design efforts in this area. It will also provide invaluable training data for emerging methods for protein design using artificial neural networks. With this advancement, future challenges that remain include developing systems for directed evolution within mammalian cells that include negative selection pressures. As synthetic biology and cell-based therapies begin to gain traction with regulators, protein designers are now very well positioned to explore this new frontier of cell–cell interaction.

## Data Availability

Any data used to generate the figures and support the text of this review are available on request made by email to the corresponding author: michael.garton@utoronto.ca
